# A study of toxicity and differential gene expression in murine liver following exposure to anti-malarial drugs: amodiaquine and sulphadoxine-pyrimethamine

**DOI:** 10.1186/1475-2875-10-109

**Published:** 2011-05-02

**Authors:** Shrawan Kumar Mishra, Prabhat Singh, Srikanta Kumar Rath

**Affiliations:** 1 Genotoxicity Laboratory, Toxicology Division, Central Drug Research Institute, CSIR, Lucknow, PIN 226 001, India

## Abstract

**Background:**

Amodiaquine (AQ) along with sulphadoxine-pyrimethamine (SP) offers effective and cheaper treatment against chloroquine-resistant falciparum malaria in many parts of sub-Saharan Africa. Considering the previous history of hepatitis, agranulocytosis and neutrocytopenia associated with AQ monotherapy, it becomes imperative to study the toxicity of co-administration of AQ and SP. In this study, toxicity and resulting global differential gene expression was analyzed following exposure to these drugs in experimental Swiss mice.

**Methods:**

The conventional markers of toxicity in serum, oxidative stress parameters in tissue homogenates, histology of liver and alterations in global transcriptomic expression were evaluated to study the toxic effects of AQ and SP in isolation and in combination.

**Results:**

The combination therapy of AQ and SP results in more pronounced hepatotoxicity as revealed by elevated level of serum ALT, AST with respect to their individual drug exposure regimen. Furthermore, alterations in the activity of major antioxidant enzymes (glutathione peroxidase, superoxide dismutase, catalase, glutathione reductase), indicating the development of oxidative stress, was more significant in AQ+SP combination therapy. cDNA microarray results too showed considerably more perturbed gene expression following combination therapy of AQ and SP as compared to their individual drug treatment. Moreover, a set of genes were identified whose expression pattern can be further investigated for identifying a good biomarker for potential anti-malarial hepatotoxicity.

**Conclusion:**

These observations clearly indicate AQ+SP combination therapy is hepatotoxic in experimental Swiss mice. Microarray results provide a considerable number of potential biomarkers of anti-malarial drug toxicity. These findings hence will be useful for future drug toxicity studies, albeit implications of this study in clinical conditions need to be monitored with cautions.

## Background

Malaria remains to be the major killer disease in the developing countries that affects lives of more than 500 million people and kills about two million of them annually [1]. Most of the drugs that are used to treat malaria can be broadly grouped into 4-aminoquinolines, 8-aminoquinolines, anti-folates, artemisinin derivatives, hydroxyl naphthoquinones and certain class of antibiotics, such as doxycycline and clindamycin. 4-aminoquiniline derivatives, such as chloroquine and amodiaquine (AQ), have been the first-line drugs against malaria for past several decades. Development of resistance against these drugs in several parts of world necessitated the use of other drugs along with it for efficient treatment. Malaria treatment guidelines issued by WHO also recommends the use of AQ and SP combinations for the treatment of chloroquine-resistant malaria [2-4]. Many clinical trials and field studies, carried primarily in African countries, showed that AQ in combination with SP was very effective in controlling cases of malaria [5-7]. Although resistance against sulphadoxine-pyrimethamine has also been reported in parts of East Africa [8-10], it remains a good choice for rest of the world, including West Africa [11]. Notwithstanding their utility in controlling malaria, most of these anti-malarials are also associated with risk of drug-induced toxicity [12-14]. In spite of wide use of AQ and SP as anti-malarials, there is dearth of scientific literature describing their potential toxicity [15].

Liver is a vital organ of body and mainly involved in drug metabolism and its biotransformation. Its unique position and crucial link with gastro-intestinal tract renders it highly vulnerable to drug induced toxicity [16,17]. Previously, Noel *et al*. had described toxicity and gene expression alterations in murine liver following exposure to the anti-relapse anti-malarial drugs primaquine [18] and bulaquine [19]. High throughput gene expression profiling facilitates prediction of toxicity and interpretation of mechanism of toxicity based on distinct gene expression changes. The simplest approach to identify genes of potential interest through several related experiments is to search for those that are consistently either up- or down-regulated [18-20]. Therefore, an attempt was made to delineate the mechanism of anti-malarial drug toxicity in liver tissue following exposure to AQ and SP combination in murine models.

## Methods

### Animal groups, drug administration and tissue collection

10-12 weeks old, male Swiss albino mice (*Mus musculus*), weighing 25-30 g (Central Drug Research Institute, Lucknow, India) were randomly assigned to control and treatment groups. All animal procedures were performed following IAEC approval (115/07/Toxicol./IAEC dated 11.9.2007) and in compliance to institutional animal ethics guidelines. The animals were acclimated to optimal conditions of temperature (25 ± 2°C) and light/dark cycle (12 h each) before initiation of drug administration. The doses for AQ and SP were calculated from human therapeutic doses [21] based on equivalent body surface area index [22]. The duration of dosing in mice was also similar to the human therapeutic regime. Animals were divided into four groups each consisting of six animals and were given following dosages orally.

Group 1: 1% DMSO-treated controls, for three consecutive days

Group 2: AQ, 120 mg/kg for three consecutive days

Group 3: 300 mg/kg sulphadoxine and 15 mg/kg pyrimethamine on day one

Group 4: 120 mg/kg AQ and SP, 300 mg/kg and 15 mg/kg respectively, on day one followed by 120 mg/kg AQ, on day two and three

All animals were sacrificed by cervical dislocation on day four of study and liver was taken out after perfusion with normal saline and a part of it is kept at -70°C until further analysis. Prior to sacrifice blood was taken out from cardiac puncture from each animal and left undisturbed for 30 minutes for serum separation. A part of liver tissue was immediately fixed in 10% formal saline for histological investigations.

### Serum biochemistry and liver histology

Alanine aminotransferase (ALT), aspartate aminotransferase (AST) [markers of hepatotoxicity] levels were estimated in the serum with automated biochemical analyzer using the kits (Beckmann). Fixed liver tissues were washed overnight, dehydrated through graded alcohols and embedded in paraffin wax. Serial sections of 5 μm thickness were stained with haematoxylin and eosin (H&E) for histological examination.

### Biochemical estimation of antioxidant enzymes in liver tissue fraction

Markers of oxidative stress {tissue levels of lipid peroxidation; LPO [a measure of malondialdehyde (MDA) concentration] and reduced glutathione level; GSH} and enzyme activities of major antioxidant enzymes (glutathione peroxidase, superoxide dismutase, catalase, glutathione reductase) were estimated in liver tissue homogenates using standard tests [23-27].

### RNA isolation, cDNA labeling and hybridization

50 mg frozen liver tissue was crushed in liquid nitrogen and immediately homogenized (Heidolph, Germany) in 1 ml of TRI reagent (Sigma, St. Louis, MO, USA) to isolate total RNA. RNA samples with approximately 2:1 ratio of 28S:18S rRNA and 260/280 values ≥ 1.8 were used for gene expression analysis. Equal amount of RNA from individuals of the same group was pooled to eliminate inter-individual variations. 25 μg of pooled RNA was converted into labeled cDNA using CyScribe First Strand cDNA-labeling kit (Amersham, Buckinghamshire, UK) following manufacturer's protocol. Labeled cDNA was purified with GFX columns as per manufacturer's guidelines and subsequently concentrated by evaporation under vacuum after estimating the percent incorporation of the dyes with a spectrophotometer (Thermo, Waltham, MA, USA). Dye swap technical replicate experiments were performed with aliquots of same RNA preparation to address inconsistencies regarding dye incorporation and other technical means of variance. The Cy5- and Cy3-labeled cDNA samples were mixed in CyScribe Hyb buffer (Amersham, Buckinghamshire, UK) containing 10 μg/ml sheared salmon sperm DNA and 10 μg/ml yeast tRNA (Ambion, Austin, Texas, USA) as blocking agents. The labeled sample was hybridized to mouse 22.4k arrays [28] for 18 h at 42°C.

### Scanning and microarray data analysis

The arrays were washed and subsequently scanned to collect raw data with Array Scanner III supported with Image-Quant version 5 (Molecular Dynamics). Intensity values were extracted from the scanned images with ArrayVision version 8 (Imaging research, GE healthcare Biosciences Corp., Piscataway, NJ, USA). Raw intensity data was analyzed with Avadis Express version 4.3 (Strand life Sciences, Bangalore, India) and the background corrected intensities were LOWESS normalized (Cy5 against Cy3) to obtain log (base 2) ratios. Furthermore, log2 values of duplicate spots were averaged in order to get a single mean value to perform *k*-means clustering with MeV version 3.1 [TM4, The Institute of Genomic Research [29]]. Each expression cluster was further clustered hierarchically with Euclidean distance matrix and average linkage to identify gene with similar expression patterns. Raw and log transformed data (series accession no. GSE 17392) has been submitted to Gene Expression Omnibus database [30] and conforms to MIAME guidelines developed by microarray gene expression data (MGED) society.

### Real time-PCR

mRNA was reverse transcribed according to the manufacturer's instruction (First Strand cDNA Synthesis Kit for RT-PCR, Invitrogen, California, USA). PCRs were performed on a Light Cycler 480 System (Roche Diagnostics) in 96-well plates. Each reaction was carried out in 20 μl reaction volume comprising of SYBR Green qPCR Master Mix (Invitrogen, California, USA), cDNA template, 200 nM of forward and reverse primers and nuclease-free water. Serial dilutions of genomic DNA (250-0.08 ng) were used to generate a quantitative PCR standard curve. The LightCycler protocol was: 2 min. of UDG incubation (Invitrogen, California USA) at 50°C followed by 10 min. of 95°C hot-start enzyme activation; 40 cycles of 95°C denaturation for 15 s, 60°C annealing and elongation. Melting curve analysis temperatures were 95°C for 5 s, 70°C for 60 s, and then heating to 95°C. Water was used as the template for negative control amplifications included with each PCR. Data were analyzed using the Roche LightCycler 480 software and Cp was calculated by the Second Derivate Maximum Method [31]. The amount of the target mRNA was examined and normalized to the GAPDH gene mRNA. The relative expression ratio of a target gene was calculated as described by Pfaffl [32], based on real-time PCR efficiencies. Results reported were obtained from at least three biological replicates and PCR runs were repeated at least twice.

### Statistical analysis

Data were expressed as the mean ± standard error of the means (S.E.M.). Group means were compared by one-way analysis of variance (ANOVA) with Newman-Keuls post analysis test. The differences in the data obtained were considered statistically significant when the P-value was less than 0.05. All statistical analysis was done through using Prism ver.5 (GraphPad Software Inc., USA).

## Results

### Effect of amodiaquine and sulphadoxine-pyrimethamine treatment on the biomarkers of hepatotoxicity and oxidative stress

**Figure 1:**
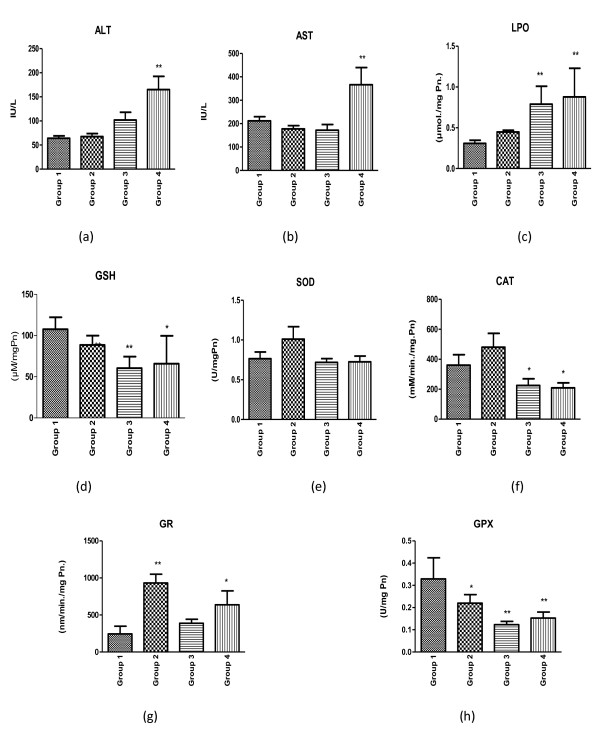
**(a - h) Assessment of markers of hepatotoxicity and oxidative stress following exposure of AQ, SP and AQ+SP**. Group 1: untreated control; Group 2: treated with AQ, 120 mg/kg body wt[AQ]; Group 3: treated with sulphadoxine (300 mg/kg) and pyrimethamine (15 mg/kg) [SP]; Group 4: Co-treatment of AQ and SP[AQ+SP]; [*(P < 0.05), ** (P < 0.01), *** (P < 0.001)].

Treatment of AQ at 120 mg/kg does not impart hepatotoxicity or oxidative stress, as levels of ALT, AST, LPO and GSH were comparable to that of untreated control. Although administration of SP does not cause any elevation in level of ALT or AST, it causes appreciable oxidative stress, as a significant elevation in LPO and a decrease in GSH were observed in mice dosed with SP. Interestingly, co-administration of AQ and SP (i.e. AQ+SP) causes both hepatotoxicity as well as oxidative stress as evident from marked increase in ALT, AST, LPO and decrease in GSH (Figure 1a, b, c and 1d).

### Effect of amodiaquine and sulphadoxine-pyrimethamine treatment on antioxidant enzymes in liver tissue fraction

Effects of AQ and SP treatment on enzymatic activities of SOD, catalase, GR and GPx, which are the major antioxidant enzymes in liver tissue fraction, were investigated. SOD activity was not altered after AQ and SP treatment, while catalase and GPx activities were drastically reduced by the treatment of SP and AQ+SP. However, AQ administration did cause a moderate, statistically non-significant, increase in the activity of SOD and catalase. However, activity of GR was increased by administration of AQ and AQ+SP combination (Figure 1e, f, g and 1h).

### cDNA Microarray analysis of differential gene expression in murine liver and kidney exposed to anti-malarials amodiaquine and sulphadoxine-pyrimethamine

Following AQ administration in murine liver, a total of 133 probes were differentially regulated, of which 60 were up-regulated and 73 down-regulated. Some of these are listed in Table 1. Major important up-regulated probes following AQ dosing included the TAP binding gene involved in antigen processing, the neogenin gene involved in ATP binding, the dihydropyrimidinase like 5 gene involved in axon guidance, the ankyrin repeat domain 6 gene involved in DNA binding and genes for GATA binding protein 2 involved in DNA binding and transcription. Some of the important down-regulated probes following AQ administration included the DEAD box polypeptide 6 gene involved in ATP-dependent helicase activity, the voltage dependent calcium channel L type alpha 1 C subunit gene involved in calcium channel activity, the lipoma HMGIC fusion partner-like 2 gene involved in general metabolism, and the GCN5 gene involved in N-acetyl transferase activity.

**Table 1:** List of important differentially expressed probes after administration of AQ in murine Liver.

Spot labels	Fold Change	Gene Name/Description	GO : Biological function
11544	2.27	TAP binding protein	antigen processing
20523	2.73	RAB39B, member RAS oncogene family	GTP binding
18680	2.49	Neogenin	ATP binding
11260	2.18	ATPase, Ca^++ ^transporting, plasma membrane 1	ATP binding
12910	2.20	Dihydropyrimidinase-like 5	axon guidance
18325	3.18	Calcium channel, voltage-dependent, L type, alpha 1D subunit	calcium channel activity
9921	3.51	Ankyrin repeat domain 6	DNA binding
18064	6.11	GATA binding protein 2	DNA binding
20032	2.51	Parathyroid hormone receptor 1	G-protein coupled receptor activity
12886	3.62	Transmembrane protein with EGF-like and two follistatin-like domains 1	growth factor activity
21365	3.33	Solute carrier family 38, member 1	L-glutamine transport
492	6.38	DNA segment, Chr 5, Wayne State University 178, expressed	phospholipid biosynthesis
22462	-4.53	DEAD (Asp-Glu-Ala-Asp) box polypeptide 6	ATP-dependent helicase activity
10941	-2.32	Calcium channel, voltage-dependent, L type, alpha 1C subunit	calcium channel activity
2989	-2.69	Nucleoporin 153	DNA binding
22225	-4.12	Suppressor of variegation 4-20 homolog 1 (Drosophila)	histone lysine N-methyltransferase activity
15707	-2.09	Lipoma HMGIC fusion partner-like 2	metabolism
9653	-2.55	GCN5 general control of amino acid synthesis-like 2 (yeast)	N-acetyltransferase activity
7818	-2.15	Nuclear receptor subfamily 3, group C, member 2	transcription factor activity

Fold change (FC) >2(Up-regulated) and FC<-2 (Down-regulated) and P < 0.01.

Administration of SP in murine liver leads to differential regulation of 156 probes of which 90 were up-regulated and 66 down-regulated, some of which are listed in Table 2. Some of the important up-regulated probes following SP treatment included the DEAH box polypeptide 15 gene involved in ATP-dependent helicase activity, the transketolase gene involved in calcium ion binding, the procollagen type VI alpha 2 genes mainly involved in cell adhesion, the procollagen lysine 2-oxoglutarate 5-dioxygenase 2 genes involved in endopeptidase inhibitor activity and a gene coding for RNA binding motif protein X. Major down-regulated probes following SP administration included the CDC42 effector protein 1 (Rho GTPase binding) involved in signal transduction, the serine (or cysteine) peptidase inhibitor clade B member 6a gene involved in endopeptidase inhibitor activity and the cytochrome c oxidase subunit VIb polypeptide 2 gene involved in electron transfer.

**Table 2:** List of Important differentially expressed probes after administration of SP in murine Liver.

Spot labels	Fold Change	Gene Name/Description	GO : Biological function
5638	2.01	Histone deacetylase 9	Histone deacetylase activity
728	2.04	DEAD (Asp-Glu-Ala-Asp) box polypeptide 42	ATP binding
15419	2.33	DEAH (Asp-Glu-Ala-His) box polypeptide 15	ATP-dependent helicase activity
1745	2.66	Transketolase	Calcium ion binding
14698	2.76	Procollagen, type VI, alpha 2	Cell adhesion
17252	2.02	Procollagen lysine, 2-oxoglutarate 5-dioxygenase 2	Endopeptidase inhibitor activity
2107	2.62	Lymphocyte antigen 6 complex, locus G6C	Extracellular space
14620	2.11	RNA binding motif protein, X chromosome retrogene	RNA binding
21645	2.11	RNA binding motif protein 28	RNA binding
12990	2.11	Eukaryotic translation initiation factor 1	Translation factor activity
21844	-3.44	CDC42 effector protein (Rho GTPase binding) 1	Signal transduction
8922	-2.20	Serine (or cysteine) peptidase inhibitor, clade B, member 6a	Endopeptidase inhibitor activity
5874	-7.13	Cytochrome c oxidase subunit VIb polypeptide 2	Unknown
6344	-5.56	Tumor protein D52	-------------
11499	-2.21	Unknown	-------------
11550	-2.54	RIKEN cDNA 4930471M23 gene	-------------
11891	-2.26	TBC1 domain family, member 19	-------------

Fold change (FC) >2(Up regulated) and FC<-2 (Down regulated) and P < 0.01.

Co-administration of AQ and SP for three consecutive days resulted in differential regulation of 231 probes, including 118 up-regulated and 113 down-regulated probes (Table 3). Major up-regulated probes following co-exposure of AQ and SP included genes having a cysteine and histidine-rich domain (CHORD) containing zinc-binding protein 1, mainly involved in calcium ion binding, the solute carrier family 28 (sodium-coupled nucleoside transporter) member 3 gene involved in ion transport, the Kelch-like 2 Mayven (Drosophila) gene involved in actin binding, the integrin beta 8 gene involved in cell adhesion, the gene for suppression of tumorigenicity (colon carcinoma) involved in cell migration and mortality factor 4 like 1 gene involved in cell proliferation. Some of the major down-regulated probes following AQ+SP treatment were the lysophospholipase 3 gene involved in acyltransferase activity, the mitogen-activated protein kinase 14 gene involved in ATP binding, the transforming growth factor beta receptor I gene involved in ATP binding, the procollagen type VI alpha3 gene involved in cell adhesion, the gene for microfibrillar-associated protein 4 involved mainly in cell adhesion, the BTB and CNC homology 2 genes involved in DNA binding and the CXXC finger 1 (PHD domain) gene also involved in DNA binding.

**Table 3:** List of Important differentially expressed probes after administration of AQ+SP in murine Liver.

Spot labels	Fold Change	Gene Name/Description	GO: Biological Function
3557	2.03	Cysteine and histidine-rich domain (CHORD)-containing, zinc-binding protein 1	Calcium ion binding
1410	3.02	Solute carrier family 28 (sodium-coupled nucleoside transporter), member 3	Integral to plasma membrane
16564	2.50	Kelch-like 2, Mayven (Drosophila)	Actin binding
5639	2.51	Integrin beta 8	Cell adhesion
10049	2.49	Suppression of tumorigenicity 14 (colon carcinoma)	Cell migration
21956	2.25	Mortality factor 4 like 1	Cell proliferation
14809	2.61	Zinc finger, SWIM domain containing 4	Cellular component
21155	2.42	Metal response element binding transcription factor 1	DNA binding
21758	3.61	Regulatory factor X, 3 (influences HLA class II expression)	DNA binding
1649	5.71	Polymerase (RNA) III (DNA directed) polypeptide F	DNA binding
20006	3.53	Cytochrome b5 type B	Electron transport
12949	2.47	GTP binding protein (gene overexpressed in skeletal muscle)	GTP binding
492	2.47	DNA segment, Chr 5, Wayne State University 178, expressed	Integral to membrane
11468	3.82	DNA segment, Chr 18, ERATO Doi 653, expressed	Integral to membrane
9206	2.18	Dystrobrevin binding protein 1	Muscle development
12725	3.03	Dolichyl-di-phosphooligosaccharide-protein glycotransferase	N-linked glycosylation via asparagine
14957	3.64	Adenosine deaminase, RNA-specific, B2	RNA binding
15208	2.70	Solute carrier family 25 (mitochondrial carrier, glutamate), member 22	Transporter activity
22601	3.18	Ubiquitin-conjugating enzyme E2D 2	Ubiquitin-dependent protein catabolism
10183	-2.83	Lysophospholipase 3	Acyltransferase activity
6999	-3.22	Mitogen activated protein kinase 14	ATP binding
22383	-2.63	Transforming growth factor, beta receptor I	ATP binding
2877	-2.03	Procollagen, type VI, alpha 3	Cell adhesion
18522	-3.11	Microfibrillar-associated protein 4	Cell adhesion
874	-3.24	BTB and CNC homology 2	DNA binding
11690	-8.17	CXXC finger 1 (PHD domain)	DNA binding
13862	-2.05	GLI-Kruppel family member GLI3	DNA binding
19141	-2.44	Protein disulfide isomerase associated 6	DNA binding
17585	-2.07	AT rich interactive domain 5B (Mrf1 like)	DNA binding
18686	-3.25	Cytochrome b5 type B	Electron transport
16362	-3.08	Proteasome (prosome, macropain) subunit, beta type 2	Endopeptidase activity
20020	-4.67	Phosphatidylinositol 3-kinase, C2 domain containing, alpha polypeptide	Glycerophospholipid metabolism
13958	-2.83	RAS related protein 1b	GTP binding
11531	-3.27	Zinc metallopeptidase, STE24 homolog (S. cerevisiae)	Hydrolase activity
13203	-2.64	SH2-B PH domain containing signaling mediator 1	Intracellular signaling cascade
19040	-2.26	Malate dehydrogenase 2, NAD (mitochondrial)	Malate dehydrogenase activity
5575	-2.06	Ring finger protein (C3HC4 type) 19	Protein ubiquitination
6605	-3.30	Cleavage and polyadenylation specific factor 4	RNA binding

Fold change (FC) >2(Up regulated) and FC<-2 (Down regulated) and P < 0.01.

Real time quantitative PCR analysis showed that most of genes that are differentially expressed in microarray produced similar results in PCR too, i.e. the genes which are up-regulated in microarray are up-regulated in real time PCR too and *vice versa *(Table 4).

**Table 4:** List of genes with their description and expression results by Q-PCR and microarray following treatment with AQ and SP in murine liver.

Gene Symbol	Gene Name/Description	Q-PCR fold change	Microarray Result Up regulated(▲)/Down regulated (▼)
ADRA1B	Adrenergic receptor, alpha 1b	-6	▲
CYP1A2	Cytochrome P450, family 1, subfamily a, polypeptide 2	-6	▼
CYP2E1	Cytochrome P450, family 2, subfamily e, polypeptide 1	-5	▼
SC4MOL	Sterol-C4-methyl oxidase-like	-4	▼
H2DM	Histocompatibility 2, class II,	1.2	▲
RAC	RAS-related C3 botulinum	-1.8	▼
MCM4	Minichromosome maintenance deficient 4 homolog	5	▲
VKORC	Vitamin K epoxide reductase complex,	-1.5	▼
SC5D	Sterol-C5-desaturase (fungal ERG3, delta-5-desaturase) homolog	7	▲
ADH1	Alcohol dehydrogenase 1 (class I)	4.8	▲
GADD45	Growth arrest and DNA-damage-inducible 45 gamma	3.2	▲
UGT2B1	UDP glucuronosyltransferase 2 family, polypeptide B1	5	▲
MCM5	Minichromosome maintenance deficient 5 homolog	2.5	▲
GCGR	Glucagon receptor	8	▲

Note the direction similarity among Q-PCR and microarray findings for gene expression results.

## Discussion

Amodiaquine and sulphadoxine-pyrimethamine offer a great potential as effective anti-malarial against chloroquine-resistant malaria and has been used in many parts of Africa as first-line anti-malarial treatment. However, considering the previous history of drug-induced hepatitis, oxidative stress associated with these drugs particularly AQ, it becomes imperative to study the toxicity associated with these drugs and their combination in liver tissue.

**Figure 2:**
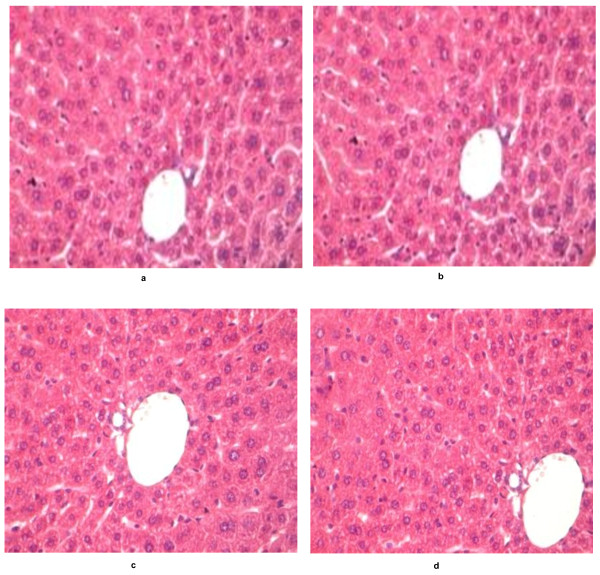
**Murine liver cross-sections treated with amodiaquine and sulphadoxine: (a) untreated control, (b) treated with 120 mg/kg of AQ, (c) treated with 300 mg/kg of sulphadoxine and 15 mg/kg of pyrimethamine (SP), (d) Co-exposure of 120 mg/kg AQ and 300 mg/kg sulphadoxine along with 15 mg/kg of pyrimethamine (AQ+SP)**.

Dosages and duration of AQ and SP treatment in Swiss mice was according to the human therapeutic equivalent dose and malaria treatment regimen suggested by WHO guidelines [4]. This observation was that only co-treatment of AQ and SP (AQ+SP) as recommended combination therapy regimen produces toxicity and not their individual exposure. However, treatment with SP alone does produces appreciable oxidative stress leading to a conclusion that observed hepatotoxicity and oxidative stress in AQ+SP group might be a result of either SP toxicity alone or an additive effect of both these drugs. Interestingly, none of these drugs or drug combinations results in alterations in normal liver histology as no histopathological damage was observed in any sections of liver tissues (Figure 2).

Previous reports showed that anti-malarials, particularly chloroquine, produce oxidative stress in liver tissue [14], and it was also interesting to study the alteration in antioxidant profile of major antioxidant enzyme present in liver tissue fraction. Results showed that the activity of SOD was not affected either by the treatment of AQ or SP or their combination (AQ+SP). However, the level of GPx was significantly reduced in all three treatment groups and catalase activity was reduced in SP and AQ+SP group in murine liver fraction. The decrease in the activity of GPx observed in this study might be the result of a decrease in GSH content; a measure substrate in GPX catalyzed reaction. Interestingly, GR activity was observed to increase in AQ and AQ+SP. The alterations in activities of antioxidant enzymes of liver observed in the present study were an indication of oxidative injury brought by the AQ and SP dosing.

**Figure 3:**
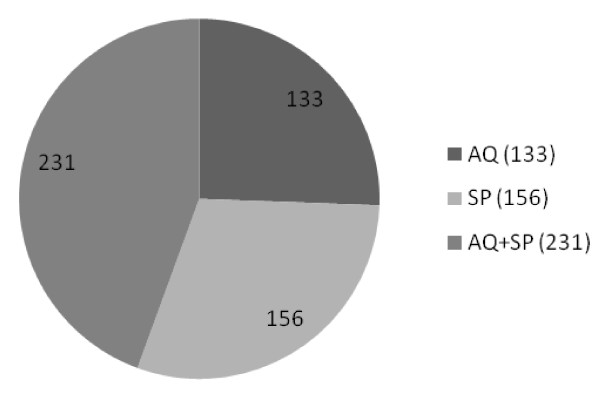
**Total number of differentially expressed genes following exposure to anti-malarial drugs in murine liver**.

High throughput expression profiling facilitates the prediction and mechanism of toxicity based on distinct gene expression changes. Therefore, the study of differential gene expression in murine liver at high statistical stringency (i.e. P < 0.01 and expression fold change >2) clearly indicated that the molecular mechanism of AQ and SP induced oxidative stress. Furthermore, validation of microarray findings using qRT-PCR further substantiates these results, which is the most sensitive and accurate method for validating microarray-based differential expression of genes [33]. The pattern of differential expression of genes in combination therapy, i.e. the AQ+SP treated groups, were on an expected line with biochemical observations, showing more robust expression pattern than either of the drug given alone. Here the number of differentially expressed probes was 231, far more differentially expressed genes than AQ (133) or SP (156) alone. Of the 231 differentially expressed genes in murine liver after AQ+SP treatment, the number of up-regulated (118) and of down-regulated (113) probes was almost similar (Figure 3).

**Figure 4:**
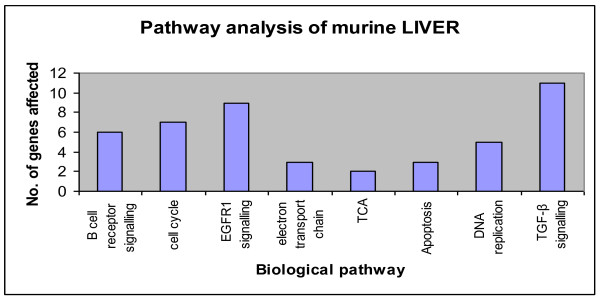
**Important biological pathways regulated by administration of all the three dose categories (AQ, SP and AQ+SP) in murine liver**.

GenMAPP and MAPPFinder tools [34] were utilized to enlist the various biological pathways that are perturbed following exposure to AQ, SP or their combination (AQ+SP). The pathways that are most affected are signaling pathways, carbohydrate metabolism, oxidative stress and drug metabolism (Figure 4). These observations suggest that anti-malarial drug exposure imparts stress in liver tissue causing changes in mRNA expression level of antioxidant pathway and major drug metabolism pathway.

One of the many genes that are up-regulated in murine liver following exposure to AQ, SP and their co-treatment i.e. AQ+SP includes EPRS (glutamyl-prolyl tRNA synthetase). EPRS is a multifunctional aminoacyl-tRNA synthetase that catalyzes the aminoacylation of glutamic acid and proline tRNA species [35]. Sampath *et al. *[36] showed that EPRS has a regulated, noncanonical activity that blocks synthesis of ceruloplasmin. Fall in the level of ceruloplasmin which is the major copper carrier protein, is an indication of hepatic stress [37], so the elevation in the level of EPRS following anti-malarial drug treatment can explain the observed hepatic stress. Supervilin (SVIL) is another gene that is consistently up-regulated in murine liver following exposure to AQ, SP and their combination. This gene codes for a protein, which is tightly associated with both actin filaments and plasma membranes, suggesting that it forms a link between the actin cytoskeleton and the membrane. An up-regulated SVIL (which is required for membrane integrity) following drug treatment may be an explanation for the rise in lipid peroxidation level observed in the present study. It appears that membrane damage following anti-malarial drug treatment is an inducing factor for up regulation of supervillin. Some of the many genes that were up-regulated in the present study include HSP90ab1, PAWR, and IKbRb among others. An up-regulated HSP90ab1 indicates that anti-malarial drug exposure has resulted in the development of hepatic stress. The PAWR genes are found to be transcriptionally induced by apoptotic signals in the rat ventral prostate [38]. Woronicz *et al. *[39] observed that IKbRb activates NF-kappa-B when overexpressed and phosphorylate serine residues 32 and 36 of I-kappa-B-alpha and 19 and 23 of I-kappa-B-beta. Therefore, upregulated PAWR and IKbRb in murine liver is an indication of cellular toxicity and inflammatory responses within liver hepatocytes following anti-malarial exposure.

One of the several genes that were down-regulated following anti-malarial exposure in murine liver is the myotubularin related protein 2 (MTMR2) gene. The MTMR2 gene encodes a protein that belongs to the myotubularin family, which is characterized by the presence of a phosphatase domain. Berger *et al. *[40] determined that mouse MTMR2 gene dephosphorylates phosphatidylinositol 3-phosphate (PI3P) and phosphatidylinositol 3, 5-bisphosphate (PI3, 5P2) with high efficiency and peak activity at neutral pH. A perturbation in phosphatidylinositol pathway resulting from down regulated MTMR2 expression is an indication of disturbances of signaling pathways following anti-malarial treatment.

## Conclusion

Both biochemical and microarray results suggest that combination therapy of AQ and SP are more damaging than their individual monotherapies. Microarray results further suggests that present anti-malarial combination therapies lead to inflammatory responses and perturbed signaling cascade leading to general hepatic stress as observed in biochemical evaluation of liver tissue. Furthermore, expression level of EPRS, SVIL, PAWR, and MTMR2 can be good markers for anti-malarial drug induced hepatotoxicity. Hence, the present study can help in understanding anti-malarial drug induced toxicity. However, the clinical implication of the study needs to be evaluated further with caution as this study in experimental mice may not hold equally good in case of malaria prophylaxis and treatment for human population.

## Competing interests

The authors declare that they have no competing interests.

## Authors' contributions

SKM Conceptual design of work, experimental work, data analysis and manuscript writing. PS Experimental work and data analysis. SKR Conceptual design of work, overall supervision of work and manuscript writing. All authors read and approved the final manuscript
